# Excited Electronic States of Sr_2_: Ab Initio Predictions and Experimental Observation of the 2^1^Σ_*u*_^+^ State

**DOI:** 10.1021/acs.jpca.3c02056

**Published:** 2023-05-16

**Authors:** Jacek Szczepkowski, Marcin Gronowski, Anna Grochola, Włodzimierz Jastrzebski, Michał Tomza, Paweł Kowalczyk

**Affiliations:** † Institute of Physics, Polish Academy of Sciences, al. Lotników 32/46, 02-668 Warsaw, Poland; ‡ Institute of Theoretical Physics, Faculty of Physics, University of Warsaw, ul. Pasteura 5, 02-093 Warszawa, Poland; § Institute of Experimental Physics, Faculty of Physics, University of Warsaw, ul. Pasteura 5, 02-093 Warszawa, Poland

## Abstract

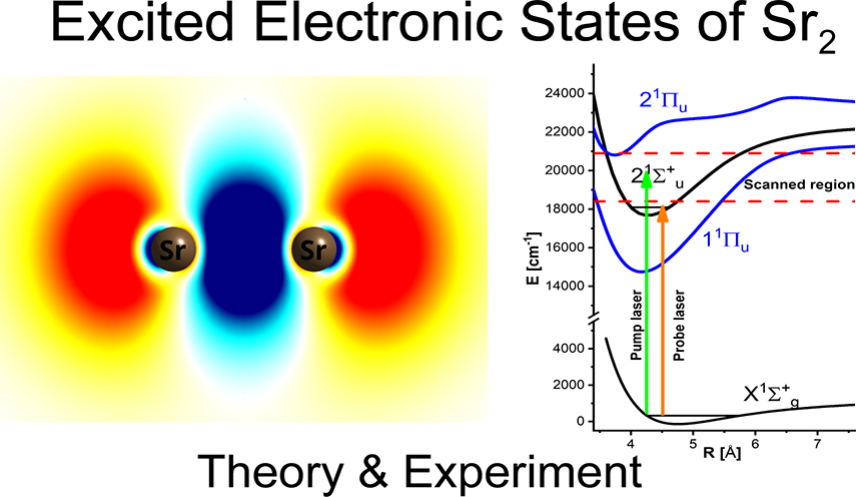

Despite its apparently simple nature with four valence electrons, the strontium dimer constitutes a challenge for modern electronic structure theory. Here we focus on excited electronic states of Sr_2_, which we investigate theoretically up to 25000 cm^–1^ above the ground state, to guide and explain new spectroscopic measurements. In particular, we focus on potential energy curves for the 1^1^Σ_*u*_^+^, 2^1^Σ_*u*_^+^, 1^1^Π_*u*_, 2^1^Π_*u*_, and 1^1^Δ_*u*_ states computed using several variants of ab initio coupled-cluster and configuration-interaction methods to benchmark them. In addition, a new experimental study of the excited 2^1^Σ_*u*_^+^ state using polarization labeling spectroscopy is presented, which extends knowledge of this state to high vibrational levels, where perturbation by higher electronic states is observed. The available experimental observations are compared with the theoretical predictions and help to assess the accuracy and limitations of employed theoretical models. The present results pave the way for future more accurate theoretical and experimental spectroscopic studies.

## Introduction

Diatomic molecules at ultralow temperatures are a perfect platform for research touching upon the very fundamentals of quantum physics and chemistry.^1^ Ultracold polar molecules have been proposed and employed for a plethora of ground-breaking experiments ranging from quantum-controlled collisions and chemical reactions^2^ to quantum simulations^3^ and precision measurements of fundamental constants and their spatiotemporal variation.^4^ After spectacular successes with alkali-metal molecules, which can be efficiently formed from ultracold atoms using magnetoassociation^5^ followed by optical stabilization,^6^ the production of ultracold molecules containing alkaline-earth-metal atoms has emerged as another important research goal.

Recently, an ultracold gas of Sr_2_ dimers in their absolute ground state was obtained using all-optical methods, where weakly bound singlet-state molecules were formed in an optical lattice by narrow-line photoassociation and transferred to the ground rovibrational level by the stimulated Raman adiabatic passage (STIRAP).^7^ Fast chemical reactions between such dimers were observed close to the universal limit. Nevertheless, ultracold Sr_2_ molecules have already been employed in a series of exciting experiments ranging from studying asymptotic physics in subradiant states^8^ to photodissociation with quantum state control.^9^ Very recently, a new type of molecular lattice clock based on ultracold Sr_2_ dimers with long vibrational coherence has also been established.^10,11^ This paves the way for upcoming applications of these molecules in quantum simulation,^12^ quantum metrology,^13^ and precision measurements probing the fundamental laws of nature.^14,15^

Exciting developments and applications of ultracold molecules described above would not have been feasible without thorough experimental spectroscopic analysis and substantial theoretical ab initio electronic structure evaluations of the underlying molecular structure. The required level of accuracy varies for each application. Generally, precise measurements can provide more accurate outcomes than theoretical calculations. However, ab initio quantum-chemical calculations of potential energy curves, permanent and transition electric dipole moments, and other molecular couplings are frequently necessary to propose, guide, and explain experimental endeavors.

Alkaline-earth-metal diatomic molecules, despite their apparently simple nature with four valence electrons and closed-shell ground electronic state, have constituted a challenge for modern electronic structure theory. Already the simplest Be_2_ dimer presents unusually strong bonding and unique shape of the ground-state potential energy curve,^16^ which accurate theoretical description required highly correlated methods.^17^ Confirming the existence of elusive vibrational states of the ground-state Mg_2_ dimer also needed state-of-the-art quantum-chemical calculations.^18^ Thus, it is not surprising that the accurate theoretical description of the Sr_2_ dimer in the ground and excited electronic states may require careful treatment, similar to lighter neutral dimers or charged Sr_2_^+^ molecular ion.^19^

The ground *X*^1^Σ_*g*_^+^ and excited 2^1^Σ_*u*_^+^ and 3^1^Π_*u*_ electronic states of Sr_2_ were initially investigated experimentally with absorption and laser-induced fluorescence spectroscopy,^20−22^ followed by high-resolution Fourier-transform laser-induced fluorescence spectroscopy of the *X*^1^Σ_*g*_^+^ state^23,24^ and the minimum region of the excited 1^1^Σ_*u*_^+^, 1^1^Π_*u*_, and 2^1^Σ_*u*_^+^ states.^25^ Recently, highly accurate measurements with ultracold Sr_2_ allowed for improving the accuracy of rovibrational spectra of the *X*^1^Σ_*g*_^+^ and 1^1^Σ_*u*_^+^ states.^7^ The ground and excited electronic states of Sr_2_ were also investigated theoretically using different computational approaches, including large-core semiempirical pseudopotentials,^26,27^ small-core relativistic pseudopotentials,^28^ and all-electron relativistic Hamiltonian.^29^ The challenging character of calculations for excited molecular electronic states could be seen in contradictory dissociation energies for the lowest-excited 1^1^Σ_*u*_^+^ and 1^1^Π_*u*_ states reported without detailed estimates of computational uncertainties. Several other studies focused solely on the ground *X*^1^Σ_*g*_^+^ electronic state,^30−35^ which is already well understood.

In this work, we investigate the excited electronic states of the Sr_2_ molecule. We start with the computational evaluation of the complete molecular electronic spectrum up to the excitation energy of around 25000 cm^–1^. Next, we compute potential energy curves for the 1^1^Σ_*u*_^+^, 2^1^Σ_*u*_^+^, 1^1^Π_*u*_, 2^1^Π_*u*_, and 1^1^Δ_*u*_ states using several variants of advanced ab initio methods. New experimental measurements of the excited 2^1^Σ_*u*_^+^ state using polarization labeling spectroscopy are presented, extending the range of observed vibrational levels to higher energies. The corresponding Dunham coefficients and experimental potential energy curve are reported. The observed perturbations in the recorded spectrum give preliminary information on higher-excited electronic states. The comparison of the experimental observations with several theoretical predictions helps to assess and benchmark the accuracy and limitations of employed theoretical models.

## Electronic Structure Calculations

### Computational Methods

Several computational approaches were used in the electronic structure calculations to assess and benchmark their accuracy. The all-electron computations employed the eXact-2-Component Hamiltonian^36^ and the equation-of-motion coupled cluster method^37^ with single and double excitations (EOM-CCSD)^38^ with the relativistic correlation-consistent core–valence quadruple-ζ basis sets (aug-cc-pwCVQ-X2C)^39^ in the version implemented in the Molpro 2022.1 program.^40^ We explored the impact of the core–electron correlation on the results by correlating only valence electrons (denoted as x2cCCSDv), valence and 4*s*4*p* electrons (denoted as x2cCCSDbc), as well as valence, 4*s*4*p*, and 3*s*3*p*3*d* electrons (denoted as x2cCCSDsc).

Other equation-of-motion coupled cluster computations used the small-core relativistic energy-consistent ECP28MDF pseudopotential^41,42^ with the quadruple- and quintuple-zeta pseudopotential-based correlation-consistent polarized core–valence basis sets (aug-cc-pCVQZ-PP and aug-cc-pCV5Z-PP, donated as QZ and 5Z, respectively)^39^ in the Cfour 2.1 software.^43^ We obtained the complete basis set (CBS) limit with two-point 1/X^3^ extrapolation.^44^ To estimate the role of the higher excitations, we compared EOM-CCSD (denoted as ecpCCSD) and EOM-CCSDT-3^45,46^ (denoted as ecpCCSDT3).

We also performed multireference computations with the standard Davidson correction.^47^ We described the valence-electron correlation by the multiconfiguration reference internally contracted configuration interaction method^48−50^ with the active space composed of 20 (sMRCI+Q) or 24 (MRCI+Q) orbitals. Such a sizable active space is necessary to correctly describe the 2^1^Π_*u*_ state of Sr_2_. The orbitals were optimized at the complete active space self-consistent field (CASSCF) level.^51^ Additionally, we employed the hybrid CIPT2+Q method,^52^ which adds the core–electron correlation to MRCI+Q by the multireference Rayleigh–Schrödinger second-order perturbation theory. All multireference computations used the aug-cc-pwCV5Z-PP basis set^39^ and were performed with the Molpro 2022.1 program. Since we had a problem converging calculations for monomers in the dimer basis set, we assumed that the basis set superposition error for multireference methods is the same as for ecpCCSD. This assumption is well justified as the total basis set superposition error is relatively small for Sr_2_.

We shifted the computed interaction energies by the sum of the appropriate experimentally measured atomic excitation energies from the NIST database^53^ and 1081.64 cm^–1^, corresponding to the molecular ground state’s depth.^24^ This procedure guarantees that the reported energies are relative to the ground state’s minimum and tend to the corresponding atomic values in their asymptotes.

### Theoretical Results

**Figure 1:**
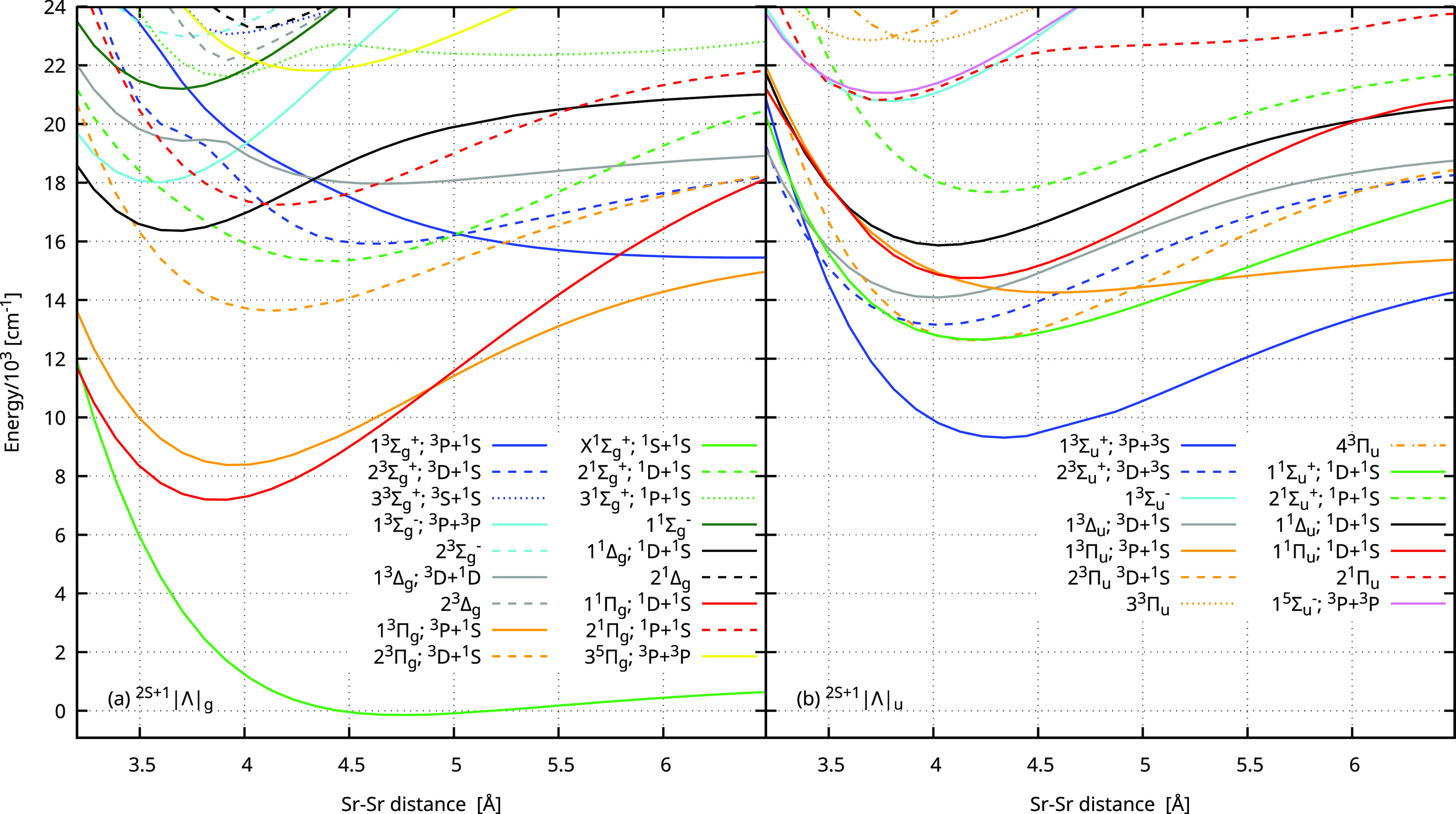
Potential energy curves for the ground and excited electronic states of Sr_2_ obtained in the nonrelativistic spin-free sMRCI+Q/5Z computations with the scalar-relativistic small-core pseudopotential. The states are labeled by symmetry and asymptote or only by symmetry in the case of states involving asymptotes for which numerical difficulties prevent obtaining whole PECs.

Figure 1 shows an overview of potential energy curves (PECs) computed with the sMRCI+Q/5Z approach, which is often the method of choice to study the excited electronic states of diatomic molecules. However, in the case of Sr_2_, this approach has some shortcomings, as we shall discuss later. The main conclusion from the overview of excited states is that the 2^1^Σ_*u*_^+^ state is fairly well separated from other states, and, thus, a relatively small number of perturbations from other states should be expected.

The strontium dimer contains 76 electrons. Therefore, a precise quantum-mechanical description is challenging. Additionally, the large charge of its nuclei limits the applicability of nonrelativistic quantum mechanics. Thus, an accurate description of Sr_2_ has to include: (*i*) extensive orbital basis set, (*ii*) valence-electron correlation, (*iii*) core–electron correlation, (*iv*) scalar relativistic contribution, (*v*) spin-related relativistic effects, like fine and hyperfine couplings, and (*vi*) leading quantum electrodynamic corrections. However, it is currently only feasible to simultaneously account for some of these effects for many-electron molecules. Here, we neglect spin-related and quantum electrodynamic contributions (*v* and *vi*) and explore the sensitivity of the potential energy curves to the remaining contributions (*i*–*iv*), which are usually the most crucial for reaching quantitative description of any molecule. The spin–orbit coupling, the largest neglected contribution, can be perturbatively added in the next steps.^28,54^

**Figure 2:**
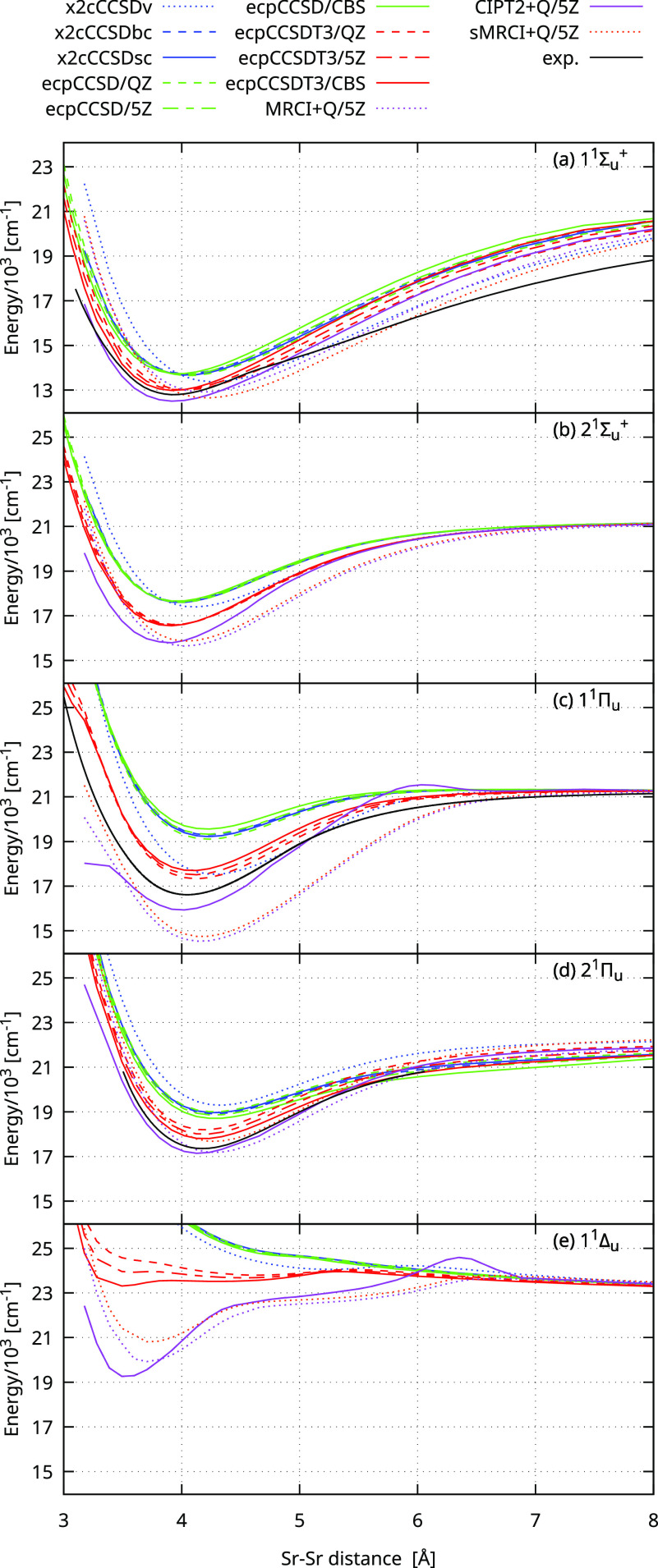
Potential energy curves for the 1^1^Σ_*u*_^+^, 2^1^Σ_*u*_^+^, 1^1^Π_*u*_, 2^1^Π_*u*_, and 1^1^Δ_*u*_ electronic states of Sr_2_ obtained with different computational methods. See the text for details. Thre potentials for 1^1^Σ_*u*_^+^, 2^1^Σ_*u*_^+^, and 1^1^Π_*u*_ are compared with the experimental curves from the present work and ref (25).

Figure 2 presents the PECs for the 1^1^Σ_*u*_^+^, 2^1^Σ_*u*_^+^, 1^1^Π_*u*_, 2^1^Π_*u*_, and 1^1^Δ_*u*_ electronic states obtained at several different levels of theory. Corresponding spectroscopic parameters are collected in Table 1. We selected these singlet ungerade states for detailed computational tests because they are the most relevant for parallel spectroscopic measurements.

**Table 1:** Origin of the State *T*_*e*_ with Respect to the Minimum of the Ground Electronic State, Dissociation Energy *E*_*e*_, Equilibrium Bond Length *R*_*e*_, Harmonic Frequency ω_*e*_, Equilibrium Rotational Constant *B*_*e*_, and Distortion Constant *D*_*e*_ for Selected States of Sr_2_, as Predicted by Various Quantum Chemical Computations^a^

state	method	*T*_e_ (cm^–1^)	*E*_e_ (cm^–1^)	*R*_e_ (Å)	ω_e_ (cm^–1^)	*B*_e_ (cm^–1^)	*D*_e_ (10^–9^ cm^–1^)
1^1^Σ_*u*_^+^	ecpCCSDT3/QZ	13013	8219	4.035	74.94	0.02356	9.32
1^1^Σ_*u*_^+^	ecpCCSDT3/5Z	13010	8221	3.982	78.17	0.02419	9.27
1^1^Σ_*u*_^+^	ecpCCSDT3/CBS	12984	8247	3.928	81.54	0.02486	9.24
1^1^Σ_*u*_^+^	ecpCCSD/5Z	13693	7538	4.017	76.41	0.02376	9.20
1^1^Σ_*u*_^+^	sMRCI+Q/5Z	12649	8582	4.209	71.25	0.02164	7.99
1^1^Σ_*u*_^+^	MRCI+Q/5Z	12910	8321	4.181	71.12	0.02194	8.36
1^1^Σ_*u*_^+^	CIPT2+Q/5Z	12502	8729	3.93	77.82	0.02484	10.0
1^1^Σ_*u*_^+^	x2cCCSDv	13373	7858	4.272	69.86	0.02102	7.61
1^1^Σ_*u*_^+^	x2cCCSDbc	13731	7500	4.037	75.28	0.02354	9.20
1^1^Σ_*u*_^+^	x2cCCSDsc	13673	7559	4.044	74.81	0.02345	9.22
1^1^Σ_*u*_^+^	theory^26^	12363		3.850	79	0.0259	
1^1^Σ_*u*_^+^	theory^27^		5490	4.01	80.21		
1^1^Σ_*u*_^+^	theory^29^	17269	5475	4.02	88		
1^1^Σ_*u*_^+^	theory^28^		8433	3.99			
1^1^Σ_*u*_^+^	exp.^25^	12796(2)		3.95(1)	80.71(3)	0.024794(2)	
1^1^Δ_*u*_	ecpCCSDT3/QZ	16605	4627	3.939	85.34	0.02472	8.30
1^1^Δ_*u*_	ecpCCSDT3/5Z	16580	4652	3.921	85.74	0.02494	8.44
1^1^Δ_*u*_	ecpCCSDT3/CBS	16550	4681	3.903	86.34	0.02518	8.56
1^1^Δ_*u*_	ecpCCSD/5Z	17626	3605	3.974	80.18	0.02429	8.92
1^1^Δ_*u*_	sMRCI+Q/5Z	15864	5367	4.041	85.22	0.02349	7.14
1^1^Δ_*u*_	MRCI+Q/5Z	15646	5585	4.031	84.99	0.02361	7.28
1^1^Δ_*u*_	CIPT2+Q/5Z	15786	5446	3.902	113.1	0.02519	5.00
1^1^Δ_*u*_	x2cCCSDv	17393	3839	4.106	78.01	0.02275	7.74
1^1^Δ_*u*_	x2cCCSDbc	17635	3596	3.982	79.82	0.02418	8.88
1^1^Δ_*u*_	x2cCCSDsc	17595	3636	3.98	80.09	0.02421	8.84
1^1^Δ_*u*_	theory^26^	16158		3.868	82	0.0257	
1^1^Π_*u*_	ecpCCSDT3/QZ	17331	3901	4.123	84.6	0.02256	6.42
1^1^Π_*u*_	ecpCCSDT3/5Z	17510	3722	4.107	84.67	0.02274	6.56
1^1^Π_*u*_	ecpCCSDT3/CBS	17695	3536	4.089	84.79	0.02293	6.71
1^1^Π_*u*_	ecpCCSD/5Z	19324	1907	4.217	75.01	0.02157	7.13
1^1^Π_*u*_	sMRCI+Q/5Z	14741	6490	4.173	85.77	0.02203	5.81
1^1^Π_*u*_	MRCI+Q/5Z	14530	6701	4.159	85.96	0.02217	5.90
1^1^Π_*u*_	CIPT2+Q/5Z	15933	5298	3.992	89.6	0.02407	6.94
1^1^Π_*u*_	x2cCCSDv	17530	3701	4.278	80.66	0.02096	5.66
1^1^Π_*u*_	x2cCCSDbc	19299	1932	4.226	75.57	0.02148	6.94
1^1^Π_*u*_	x2cCCSDsc	19219	2012	4.223	75.88	0.0215	6.91
1^1^Π_*u*_	theory^26^	16243		3.952	96	0.0246	
1^1^Π_*u*_	theory^29^	18658	4081	3.93	72		
1^1^Π_*u*_	exp.^25^	16617.86(2)		4.0473(2)	86.300(3)	0.023415(2)	6.943
2^1^Σ_*u*_^+^	ecpCCSDT3/QZ	18193	4587	4.192	82.78	0.02183	6.07
2^1^Σ_*u*_^+^	ecpCCSDT3/5Z	18000	4780	4.19	82.17	0.02185	6.18
2^1^Σ_*u*_^+^	ecpCCSDT3/CBS	17797	4983	4.187	81.52	0.02187	6.30
2^1^Σ_*u*_^+^	ecpCCSD/5Z	18858	3922	4.276	71.96	0.02097	7.13
2^1^Σ_*u*_^+^	sMRCI+Q/5Z	17674	5106	4.273	82.66	0.021	5.42
2^1^Σ_*u*_^+^	MRCI+Q/5Z	17182	5599	4.277	84.23	0.02096	5.19
2^1^Σ_*u*_^+^	CIPT2+Q/5Z	17145	5635	4.145	88.54	0.02233	5.68
2^1^Σ_*u*_^+^	x2cCCSDv	19297	3484	4.324	74.9	0.02051	6.16
2^1^Σ_*u*_^+^	x2cCCSDbc	18914	3866	4.283	72.26	0.02091	7.01
2^1^Σ_*u*_^+^	x2cCCSDsc	18959	3821	4.276	72.81	0.02097	6.96
2^1^Σ_*u*_^+^	present exp.	17358.70(10)		4.176(1)	84.2169(29)	0.021990(14)	7.12(6)
2^1^Σ_*u*_^+^	exp.^25^	17358.75(1)		4.1783(1)	84.215(1)	0.021969(1)	5.98
2^1^Π_*u*_	CCSDT-3/CBS	23846	–1066	5.715	7.442	0.01174	117
2^1^Π_*u*_	sMRCI+Q/5Z	20805	1975	3.739	104	0.02743	7.64
2^1^Π_*u*_	MRCI+Q/5Z	19921	2859	3.677	137.1	0.02836	4.86
2^1^Π_*u*_	CIPT2+Q/5Z	19241	3539	3.533	142.9	0.03074	5.69
2^1^Π_*u*_	present exp.	>19100					

a These values are compared with experimental results whenever possible. The values in parentheses are experimental uncertainties in units of last digits.

The first observation is that the PECs exhibit relatively low sensitivity to the orbital basis set size, meaning that calculations in the quadruple- and quintuple-zeta basis sets are already close enough to the complete basis set limit. This can be demonstrated by comparing the results in the quintuple-zeta basis set with the estimated CBS (ecpCCSDT3/5Z vs ecpCCSDT3/CBS). The difference in the dissociation energy is of the order of 30 cm^–1^ for the 1^1^Σ_*u*_^+^ and 1^1^Δ_*u*_ states and of the order of 200 cm^–1^ for the 1^1^Π_*u*_ and 2^1^Σ_*u*_^+^ states.

Next, we observe that correlating only valence electrons is insufficient. We systematically investigate the effect of the core–electron correlation by changing the size of the frozen core in the all-electron equation-of-motion coupled-cluster computations with the eXact-2-Component Hamiltonian (x2cCCSDv vs x2cCCSDbc vs x2cCCSDsc). The lack of the core correlation not only alters the depth of PEC by hundreds of cm^–1^, but mainly elongates the equilibrium distance. Surprisingly, the core correlation also affects the asymptotic region of the 1^1^Σ_*u*_^+^ and 2^1^Σ_*u*_^+^ states. On the other hand, the correlation of the valence and 4*s*4*p* electrons is sufficient, and the correlation of the electrons occupying the lower orbitals (replaced by the small-core pseudopotential) is not necessary.

Finally, we address the relativistic effects. Our calculations include the scalar relativistic effects only. We do not observe substantial differences between the all-electron x2cCCSDsc and small-core-pseudopotential ecpCCSD results. Therefore, we conclude that using the small-core ECP28MDF pseudopotential to account for the scalar relativistic effects is justified and sufficient, which is in agreement with other studies.^28,55^ Our calculations do not account for the spin-related part of the Dirac-Coulomb-Breit Hamiltonian. It is necessary to go beyond this approximation to correctly describe the crossing between states of different spin multiplicity coupled by spin–orbit coupling, which can be added to our curves perturbatively. For example, this coupling is important for the 1^1^Σ_*u*_^+^ state at distances larger than 4.5 Å. Kotochigova^29^ included the spin–orbit part in her computations directly, but her results for 1^1^Σ_*u*_^+^ significantly deviate from modern experimental results^25^ and our present calculations due to her approximate treatment of the electron correlation by the configuration interaction valence bond self-consistent-field approach. In contrast, Skomorowski et al.^28^ included the electron correlation directly and spin–orbit interaction perturbatively for 1^1^Σ_*u*_^+^ with the coupled cluster method and small-core pseudopotential, and obtained a much better agreement with the experiment.

Overall, the primary factor determining the accuracy of the calculations for Sr_2_ is the inclusion of high excitations in the description of the valence-electron correlation. For the analyzed electronic states, the difference between the ecpCCSDT3 and ecpCCSD results, that is, the inclusion of triple excitations, is more significant than the effect of the core–electron correlation. Among the methods used, the ecpCCSDT3 and CIPT2+Q approaches include the core–electron correlation and a substantial part of excitations higher than doubles. The CIPT2+Q method is a multireference approach that includes all possible excitations within the active space. Thus, CIPT2+Q accounts well for the static correlation but gives only an approximate description of the core–electron dynamic correlation. This approximation was necessary since an alternative approach, based on the multireference configuration interaction method with a large active space that correlates core electrons, goes beyond the technical capabilities of modern quantum-chemical programs. On the other hand, the single-reference ecpCCSDT3 approach accounts for the dynamic correlation of core and valence electrons but only includes a fraction of triple and higher excitations.

The importance of higher excitations and multireference nature can be seen by analyzing the wave functions. We inspected the squares of reference coefficients obtained with the sMRCI+Q method at *R* = 4.13 Å, close to respective equilibrium distances. We found that the electronic wave function of the 1^1^Σ_*u*_^+^ state consists mostly of single-excited determinants (72%), and the role of excitations higher than double is negligible (3%). Therefore, it is not surprising that for this state, we observe the smallest difference between energies obtained with the ecpCCSDT3 and CIPT2+Q approaches, which also agree well with another single-reference calculation reported by Skomorowski et al.^28^ The slightly smaller role of single-excited determinants is visible for the 2^1^Σ_*u*_^+^ and 1^1^Δ_*u*_ states (about 66%), where the difference between PECs calculated with the ecpCCSDT3 and CIPT2+Q methods is larger. Still, the role of triple excitations for these states is below 5%, and higher excitations are an order of magnitude less important. For the 1^1^Π_*u*_ state, we observe similar contributions from single- and double-excited determinants (49% and 39%), that explains the significant difference between the ecpCCSD and ecpCCSDT3 results. The role of triple and quadruple excitations for this state is relatively low, accounting for about 5% and 0.65%, respectively. Therefore, we can conclude that the application of the full configuration interaction method may be unnecessary to obtain accurate results for the 1^1^Σ_*u*_^+^, 2^1^Σ_*u*_^+^, 1^1^Δ_*u*_, and 1^1^Π_*u*_ states. We can also assume that the ecpCCSDT3 and CIPT2+Q approaches properly set the boundaries for the PEC shapes. Indeed, near the minima, the experimental PECs for the 1^1^Σ_*u*_^+^, 2^1^Σ_*u*_^+^, and 1^1^Π_*u*_ states lie between the curves obtained with the ecpCCSDT3 and CIPT2+Q methods.

The 2^1^Π_*u*_ state deserves special attention and particular comment because the variation of PECs obtained for this state with different methods is the largest, and this is the only state analyzed in which double excitations are dominant (75%). Additionally, it exhibits the highest contribution from the quadrupole excitations (2%). It corresponds to the ^1^P + ^1^S asymptote, and for large internuclear distances, it is repulsive. We can observe its two avoided crossings with other states of the same symmetry. The one at a larger interatomic separation involves a state from the ^3^*P* + ^3^*P* asymptote. The assignment of the second crossing is far more complex, as numerical difficulties prevent obtaining whole PECs for all states from the ^1^*D*(4*d*5*p*) + ^1^*S*(5*s*^2^), ^1^*P*(5*s*5*p*) + ^1^*S*(5*s*^2^), ^1^*D*(5*s*5*d*) + ^1^*S*(5*s*^2^) and ^3^*D*(5*s*4*d*) + ^3^*P*(5*s*5*p*) asymptotes. We suppose that doubly excited states, ^1^*D*(4*d*5*p*) + ^1^*S*(5*s*^2^) and ^3^*D*(5*s*4*d*) + ^3^*P*(5*s*5*p*), play a crucial role here. The equation-of-motion coupled cluster method with single and double excitations does not describe them accurately, so the PEC predicted at that level is mostly repulsive. The inclusion of some higher excitations by the ecpCCSDT3 method allows for a poor description of ^1^*D*(4*d*5*p*) state of Sr, where the excitation energy is overestimated by nearly 2000 cm^–1^ (see Table 2). The MRCI+Q/5Z method predicts the energy of ^1^*D*(4*d*5*p*) in a reasonably good agreement with the experiment. On the other hand, MRCI+Q/5Z tends to overestimate the depth of the potential for other states of Sr_2_. Additionally, our active space is too small to fully account for the ^1^*P*(5*s*5*p*) + ^1^*S*(5*s*^2^) and ^1^*D*(5*s*5*d*) + ^1^*S*(5*s*^2^) asymptotes. Overall, the expected position of the minimum in the 2^1^Π_*u*_ potential is in the wide range between 19000 cm^–1^ given by the CIPT2+Q/5Z method and 24000 cm^–1^ from the ecpCCSDT3/CBS computation above the minimum of the ground electronic state (see Table 1). This range covers the value *T*_e_ > 19100 cm^–1^, estimated based on our present experimental observations (vide infra). Our computations do not confirm the existence of the avoided crossing between 1^1^Π_*u*_ and 2^1^Π_*u*_ predicted by Boutassetta et al.^26^ We indeed observe that 1^1^Π_*u*_ and 2^1^Π_*u*_ approach each other in the repulsive part of the PECs, but a possible crossing may occur only in the region experimentally insignificant. We suppose that a small basis set, with an insufficient number of high angular momentum components, could have significantly decreased the precision of Boutassetta et al. results for highly excited states. However, their approach accounts well for static correlation and thus reproduces the general shape of the PECs. Czuchaj et al.^27^ reported PEC, which differs from our and Boutassetta et al. results. However, their active space in multireference computations was smaller than ours and did not allow for an accurate description of static correlation. We believe that the accurate description of 2^1^Π_*u*_ state is a major computational challenge. Most likely, the only way to obtain its reliable and accurate description is to use the full configuration interaction with a large basis set and proper account for the core and core–valence correlation method, which is out of the scope of this work. Therefore, for now, we must assume that the exact shape of the curve is unknown and falls somewhere between the curves predicted by the ecpCCSDT3/CBS and CIPT2+Q/5Z approaches.

**Table 2:** Excitation Energies (in cm^–1^) of Singlet Electronic States of the Sr Atom Obtained with Different Quantum Chemistry Methods

method	^1^*D* 5*s*4*d*	^1^*P* 5*s*5*p*	^1^*S* 5*s*6*s*	^1^*D* 4*d*5*p*	^1^*P* 5*s*6*p*	^1^*D* 5*s*5*d*
exp.^53^	20149.685	21698.452	30591.825	33826.899	34098.404	34727.447
x2cCCSDv	20144	20817	29076	33569	32494	33623
x2cCCSDbc	20659	22612	30982		34811	35355
x2cCCSDsc	20855	22652	30993		34837	35405
ecpCCSDT3/5Z	20489	21845	30517	35776	34150	34820
MRCI+Q/5Z	20162	20849	29095	33609		

We provide the potential energy curves for the 1^1^Σ_*u*_^+^, 2^1^Σ_*u*_^+^, 1^1^Π_*u*_, 2^1^Π_*u*_, and 1^1^Δ_*u*_ states obtained with the ecpCCSDT3/CBS and CIPT2+Q/5Z methods, which are the most accurate among the investigated apporaches, and for the states presented in Figure 1 obtained with the sMRCI+Q/5Z method in the Supporting Information accompanying this paper.

## Experiment

### Experimental Setup

The Sr_2_ molecules were produced in a three section heat-pipe oven^56^ of 1 m length filled in the central part with 15 g of strontium. The central part with a length of 20 cm was heated to 1020 °C, while external parts were maintained at 720 °C. In the case of strontium a proper circulation of the metal inside the heat-pipe is a challenge. To solve this problem, 1.5 g of metallic magnesium was added to its central section (strontium and magnesium form an alloy with substantially lower melting point than its constituents^57^). A steel mesh was placed separately in each section and the heat-pipe was filled with 15 Torr of argon buffer gas.

A polarization labeling spectroscopy (PLS) technique was employed to obtain spectra of Sr_2_ molecules. The PLS is a pump–probe experimental technique, which takes advantage of an optical anisotropy created in a chosen group of molecules in the sample to limit the number of observed spectral lines.^58^ In the present experiment a NarrowScan dye laser of a spectral line width of 0.07 cm^–1^ pumped with a XeCl excimer laser (Light Machinery) was used as a pump laser and its wavelength was scanned between 18400 and 20300 cm^–1^, covering transitions from the ground state of Sr_2_ to the upper part of the 2^1^Σ_*u*_^+^ state. As a probe laser a ring dye laser (Coherent 899, pumped with Sprout laser) was employed, and its wavelength was controlled with HighFinesse WS-7 wavemeter. The laser was working on Rhodamine 6G, what enabled tuning its light within a spectral range 16800–17600 cm^–1^. The probe laser wavelength was fixed on selected transitions from the ground X^1^Σ_*g*_^+^ state of Sr_2_ molecule to low levels of the 2^1^Σ_*u*_^+^ state known from the Supporting Information of the publication describing the bottom part of this state.^25^

Reference signals for wavenumber calibration of the molecular spectra were needed, therefore two auxiliary signals were recorded, namely transmission fringes from a Fabry-Pérot interferometer with FSR = 1 cm^–1^, and optogalvanic spectrum from argon and neon hollow-cathode lamps. This ensured that the uncertainty of wavenumbers determined in this way is below ±0.1 cm^–1^.

### Analysis of the Spectra

**Figure 3:**
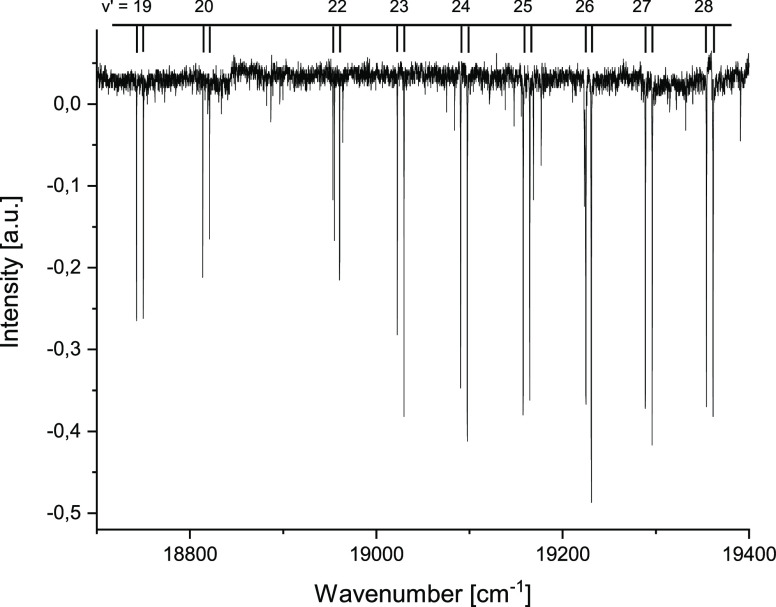
A portion of the experimental spectrum of Sr_2_ coresponding to transitions from rovibrational level *v*″ = 4, *J*″ = 90 in the ground X^1^Σ_*g*_^+^ state to consecutive rovibrational levels (*v*′ = 19–28) of the excited 2^1^Σ_*u*_^+^ state.

As the bottom part of the 2^1^Σ_*u*_^+^ state has been characterized in the Fourier-transform spectroscopy experiment by Stein et al.,^25^ we have concentrated on higher vibrational levels of this state. A typical example of the recorded spectrum of Sr_2_ is presented in Figure 3. Our experiment provided information about rovibrational levels with quantum numbers ranging from *v*′ = 13 to 52 and *J*′ from 43 to 149, solely in the most abundant ^88^Sr_2_ isotopologue. We supplemented the database with levels of ^88^Sr_2_ measured in,^25^ however to avoid too strong influence of these levels on subsequent fits of molecular constants we limited the borrowed levels to *J*′<150 and assigned them with the same accuracy of 0.1 cm^–1^ as our own data. This resulted in 714 levels taken from^25^ and 1760 levels from our own measurements. The term values of all levels were calculated by adding the measured transition energies to the energies of the initial X^1^Σ_*g*_^+^ (*v*″, *J*″) levels obtained from the highly accurate molecular constants reported in ref (23).

**Figure 4:**
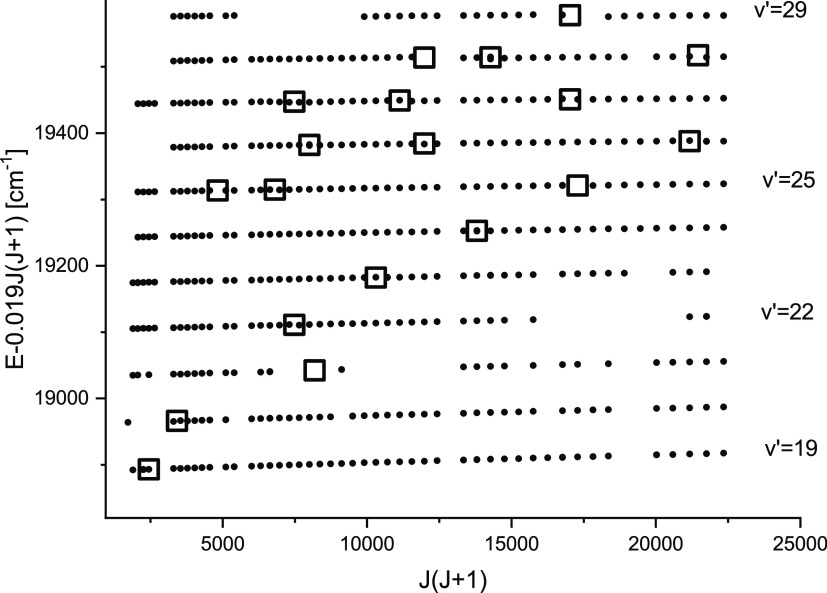
Reduced term values *E*_red_ = *E* – 0.019 × *J*(*J* + 1) cm^–1^ of part of the observed rovibrational levels in the 2^1^Σ_*u*_^+^ state (dots) plotted against *J*(*J* + 1) . The value of 0.019 cm^–1^ is an approximate rotational constant *B*_*v*_ for vibrational levels *v*′ = 19–29 displayed in the figure. Open squares indicate approximate positions of centers of perturbations. A gap in the data near *J*(*J* + 1) = 13000 is due to the lack of data on possible labeling transitions for *J* = 110–114 listed by Stein et al.^25^

**Figure 5:**
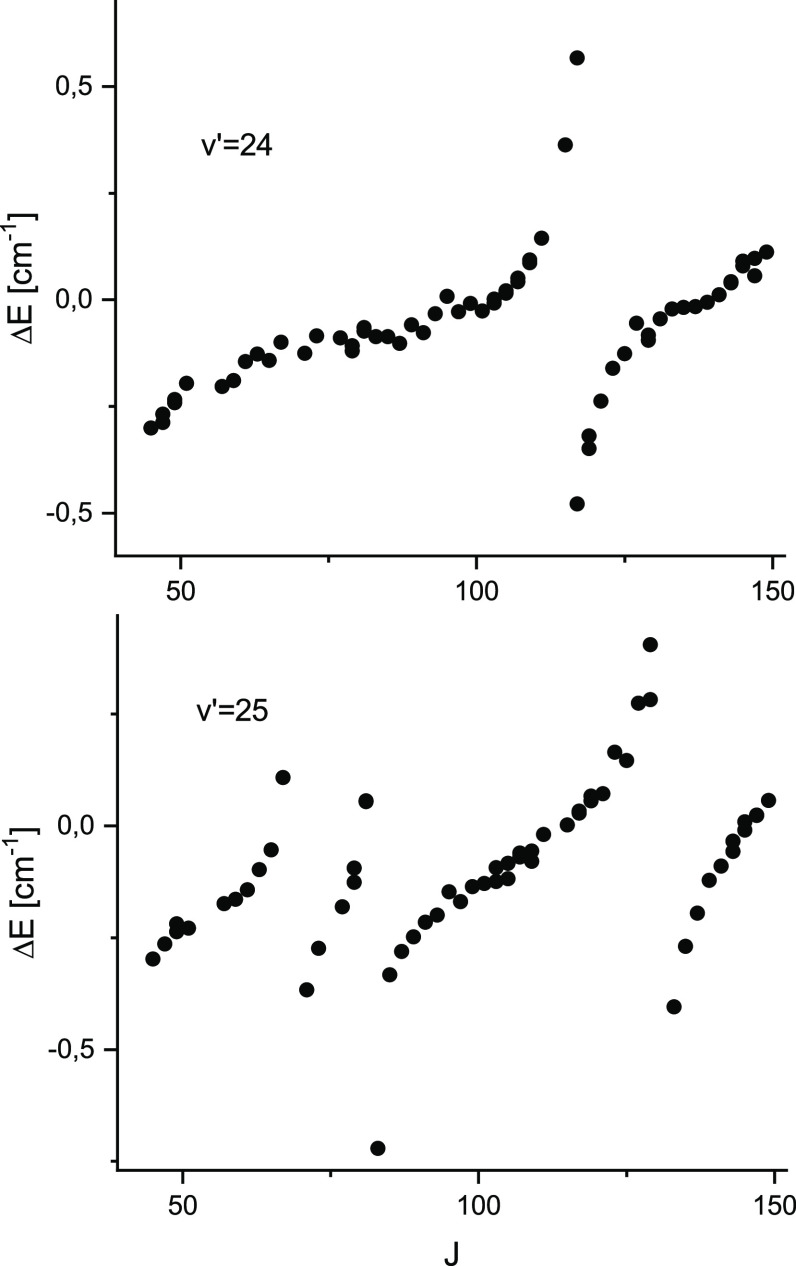
Observed shifts of the rotational energy levels in the 2^1^Σ_*u*_^+^ state from their predicted positions for vibrational levels *v*′ = 24 and 25.

Originally we tried to fit the term energies to the standard Dunham expansion
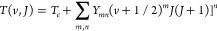
1but the rms error of the fit amounted to 0.5 cm^–1^, i.e., five times more than our experimental accuracy. This result suggested strong perturbations in the 2^1^Σ_*u*_^+^ state, particularly that the misbehaving levels, all of them corresponding to *v*′ ≳ 19, were centered around isolated (*v*′, *J*′) values and the deviations fell into patterns characteristic for perturbations. Figure 4 displays term values of several levels of the 2^1^Σ_*u*_^+^ state plotted against *J*(*J* + 1) and open squares localize regions of the observed perturbations. Figure 5 visualizes typical pattern of deviations between the observed and predicted line positions versus rotational quantum number *J*.

In the preliminary analysis presented here which aims primarily to test accuracy of theoretical predictions based on various computational methods, we decided to remove the apparently perturbed levels from the database and to fit Dunham coefficients to the remaining levels. For this purpose we originally fitted all the observed levels with various sets of Dunham coefficients and then gradually discarded these levels which differed from the expected positions by more than 0.3 cm^–1^, irrespectively to the number of coefficients used in a fit. When fitting energies of somewhat arbitrarily chosen 1636 levels (out of the total 2474) we obtained rms error 0.08 cm^–1^, this time consistent with the experimental accuracy. The Dunham coefficients have been rounded to minimize the number of digits by a procedure described by Le Roy.^59^ They are listed in Table 3 together with the equilibrium bond length *R*_e_ calculated from the rotational constant and reduced mass of strontium nuclei. A rotationless potential energy curve for the 2^1^Σ_*u*_^+^ state was constructed by a standard Rydberg-Klein-Rees (RKR) method. The vibrational term energies *G*_*v*_ and turning points *R*_–_ and *R*_+_ are given in Table 4 and the potential curve is displayed in Figure 2 along with the theoretical predictions. Our work extends the range of experimentally determined potential to 3.5 Å < *R* < 6.1 Å and more than doubles the range of covered energies.

**Table 3:** Dunham Coefficients That Describe the 2^1^Σ_*u*_^+^ State of ^88^Sr_2_ in the Range 0 ≤ *v*′ ≤ 52, *J*′ ≤ 149^a^

constant	value (cm^–1^)
*T*_e_	17358.70(10)
*Y*_10_	84.2169(27)
*Y*_20_	–0.26729(21)
*Y*_30_	–0.10850(6) × 10^–2^
*Y*_40_	–0.10760(6) × 10^–4^
*Y*_01_	0.021990(14)
*Y*_11_	–0.6808(18) × 10^–4^
*Y*_21_	–0.4860(10) × 10^–6^
*Y*_31_	–0.1030(13) × 10^–7^
*Y*_02_	–0.712(6) × 10^–8^
*R*_e_ (Å)	4.176(1)

a The numbers in parentheses give uncertainties in the last quoted digits (one standard deviation).

**Table 4:** Rotationless RKR Potential Energy Curve for the 2^1^Σ_*u*_^+^ State of Sr_2_^a^

*v*	*G*_*v*_ (cm^–1^)	*R*_–_ (Å)	*R*_+_ (Å)	*v*	*G*_*v*_ (cm^–1^)	*R*_–_ (Å)	*R*_+_ (Å)
	0	4.176					
0	42.024	4.084	4.275	26	2018.540	3.630	5.153
1	125.703	4.020	4.352	28	2150.854	3.616	5.210
2	208.838	3.978	4.408	30	2279.868	3.603	5.268
4	373.445	3.917	4.496	32	2405.468	3.590	5.327
6	535.786	3.870	4.571	34	2527.538	3.579	5.388
8	695.797	3.832	4.638	36	2645.955	3.568	5.451
10	853.411	3.799	4.701	38	2760.596	3.558	5.516
12	1008.555	3.770	4.760	40	2871.329	3.549	5.583
14	1161.155	3.744	4.818	42	2978.022	3.540	5.653
16	1311.129	3.721	4.875	44	3080.537	3.532	5.726
18	1458.395	3.700	4.930	46	3178.731	3.524	5.803
20	1602.863	3.680	4.986	48	3272.459	3.517	5.884
22	1744.442	3.662	5.041	50	3361.570	3.510	5.970
24	1883.034	3.646	5.097	52	3445.910	3.504	6.061

a The first line refers to the bottom of the potential curve, and the corresponding *R* value is the equilibrium distance. The full list of turning points of the potential is given in the Supporting Information accompanying this work.

It must be noted that in the range *v*′ = 0–18 the 2^1^Σ_*u*_^+^ state is free of (strong) perturbations which become visible only from *v*′ = 19. The most likely perturber is the 1^1^Π_*u*_ state, as the outer limb of its potential curve gradually approaches potential of the 2^1^Σ_*u*_^+^ state. However, at *v*′ = 25 apparently an additional perturbing state emerges, since perturbations become more frequent (*v*′ = 25 is perturbed at least around three *J*′ values, see Figure 5). From analysis of the theoretical potential energy curves it follows that another perturber appearing here must be the 2^1^Π_*u*_ state, the only other singlet ungerade state which is expected to be present in the vicinity. Therefore our observation indicates that the bottom of its potential cannot be located higher than approximately 19100 cm^–1^ above the minimum of the ground state potential, what can serve as another test for validity of theoretical predictions.

## Conclusion

In this study, we investigated the excited electronic states of the strontium dimer. We theoretically obtained the complete molecular electronic spectrum up to the excitation energy of around 25000 cm^–1^ using the multireference configuration interaction method. Next, we studied in detail potential energy curves for the 1^1^Σ_*u*_^+^, 2^1^Σ_*u*_^+^, 1^1^Π_*u*_, 2^1^Π_*u*_, and 1^1^Δ_*u*_ states using several advanced electronic structure methods. We evaluated the importance of the orbital basis set size, core- and valence-electron correlation, and scalar relativistic effects. Theoretical results had been motivated by our ongoing spectroscopic studies. We presented new experimental measurements of the excited 2^1^Σ_*u*_^+^ state using polarization labeling spectroscopy, extending the range of observed vibrational levels to higher energies. We reported the corresponding Dunham coefficients and experimental potential energy curve. We observed perturbations in the recorded spectrum that give preliminary information on higher-excited electronic states. We compared the available experimental observations with the theoretical predictions to assess the accuracy and limitations of employed theoretical models. Our findings provide valuable insights into the complex electronic structure of Sr_2_, paving the way for future, more accurate theoretical and experimental spectroscopic studies.

The challenging nature of excited electronic states of the Sr_2_ dimer makes them a perfect testbed and playground for near-future developments of the electronic structure theory and computation. In the following work, we plan to present calculations at the valence full configuration interaction level with large basis sets. Such converged calculations in versions with both small-core and large-core pseudopotentials and approximately included core and core–valence correlation may resolve the nature of most problematic states such as 2^1^Π_*u*_.

A more rigorous deperturbation procedure is also needed to clarify the experimental observations. It would require a coupled channels treatment involving (at least) the 2^1^Σ_*u*_^+^, 1^1^Π_*u*_ and 2^1^Π_*u*_ interacting states and such an analysis is in future plans of our group. However, more precise theoretical predictions of the relevant potential energy curves are needed to serve as a starting point for deperturbation procedure. At the present stage we show the approximate results, accuracy of which is more than sufficient for comparison with theoretical models. All the experimental term energies are listed in the Supporting Information accompanying this paper.

## Supporting Information Available

Potential energy curves of electronic states of Sr_2_ molecule calculated with the CIPT2Q5Z method (ZIP)
Potential energy curves of electronic states of Sr_2_ molecule calculated with the ecpCCSDT3/CBS method (ZIP)
Potential energy curves of electronic states of Sr_2_ molecule calculated with the sMRCI/5Z method (ZIP)
Rotationless RKR potential energy curve for the 2^1^Σ_*u*_^+^ state of Sr_2_ molecule (see Table 4) (TXT)
Experimental energies of the observed rovibrational levels in the 2^1^Σ_*u*_^+^ state (TXT)
